# Supplementary material for: Vaccine Breakthrough Infections by SARS-CoV-2 Variants after ChAdOx1 nCoV-19 Vaccination in Healthcare Workers
Source: Vaccines (Basel). 2021 Dec 31;10(1):54. doi: 10.3390/vaccines10010054 (PMC8778656; doi:10.3390/vaccines10010054)
Supplement: Supplementary file 1 [file vaccines-10-00054-s001.zip › vaccines-1522707-supplementary.pdf]

S1: Definitions used for the purpose of this study

1. *Vaccine Breakthrough infection (VBT):* A vaccine breakthrough infection was defined as the detection of SARS-CoV-2 RNA or antigen in a respiratory specimen collected from an individual who had received either one or two doses of vaccine.

2. *Partially vaccinated HCW:* The HCW who had received a single dose of vaccine or who developed infection before 14 days of the second dose was considered partially vaccinated.

3. *Fully vaccinated HCW:* The HCW who received two doses of vaccine and developed infection after 14 days of the second dose.

4. *Reinfection:* Reinfection was defined as detection of SARS-CoV-2 RNA on or after 90 days of the first detection of SARS-CoV-2 RNA and paired respiratory specimens (one from each infection episode) were available.

5. *Non-responder:* HCW with no detectable SARS COV2 IgG antibody response 14 days after the second dose of the vaccine.

6. *Low antibody levels:* SARS COV2 IgG antibodies detectable in the range of 1 to 4.62 signal/cut-off (S/CO) were considered Low level antibody response.

7. *Medium antibody levels:* SARS COV2 IgG antibodies detectable in the range of more than 4.62 to 18.45 S/CO were considered medium level antibody response.

8. *High antibody levels:* SARS COV2 IgG antibodies detectable in the range of >18.45 S/CO were considered high level antibody response.
